# New-Onset Diabetes After Transplantation in Renal Recipients: A Pilot Comparative Study of Immediate vs. Extended-Release Tacrolimus Formulation

**DOI:** 10.3390/ph18101532

**Published:** 2025-10-12

**Authors:** Ioana Adela Ratiu, Florin Bănică, Corina Moisa, Bianca Pașca, Daniela Gîtea, Iulia Dana Grosu, Gabriel Cristian Bako, Oliviu Voștinaru, Wael Abu Dayyih, Lorena Filip

**Affiliations:** 1 Faculty of Medicine and Pharmacy, University of Oradea, 1st December Square 10, 410073 Oradea, Romania; ioana.ratiu@didactic.uoradea.ro (I.A.R.); fbanica@uoradea.ro (F.B.); dgitea@uoradea.ro (D.G.); gabriel.bako@uoradea.ro (G.C.B.); 2 Nephrology Department, Emergency Clinical Hospital Bihor County, 12 Corneliu Coposu Street, 410469 Oradea, Romania; 3 Department of Internal Medicine II-Nephrology University Clinic, “Victor Babeș” University of Medicine and Pharmacy, Eftimie Murgu Sq. No.2, 300041 Timișoara, Romania; grosu.iulia@umft.ro; 4 Centre for Molecular Research in Nephrology and Vascular Disease, “Victor Babeș” University of Medicine and Pharmacy, Eftimie Murgu Sq. No.2, 300041 Timișoara, Romania; 5 Faculty of Pharmacy, University of Medicine and Pharmacy “Iuliu Hatieganu” Cluj-Napoca, Victor Babeș Street 8, 400012 Cluj-Napoca, Romania; oliviu.vostinaru@umfcluj.ro (O.V.); lfilip@umfcluj.ro (L.F.); 6 Faculty of Pharmacy, Mutah University, Al-Karak 61710, Jordan; wabudayyih@mutah.edu.jo; 7 Academy of Romanian Scientists (AOSR), 3 Ilfov Street, 050044 Bucharest, Romania

**Keywords:** tacrolimus, new-onset diabetes after transplantation, renal transplantation, extended release, immediate release, drug dissolution

## Abstract

Tacrolimus is frequently used in immunosuppressive therapy in renal transplant patients and is characterized by high toxicity, a low therapeutic index, and great individual variability. For these reasons, correct dosing is important to ensure patient safety by reducing the incidence of adverse effects while maintaining an optimal blood level that prevents graft loss. New-onset diabetes after transplantation (NODAT) affects 15–30% of patients treated with tacrolimus, with potential differences between immediate-release (IR) and extended-release (ER) formulations. **Objective**: This study seeks to compare the incidence of NODAT between IR tacrolimus and ER tacrolimus formulations in renal transplant patients and correlate it with in vitro release characteristics. **Methods**: This is a retrospective pilot study including 66 renal transplant patients (33 IR tacrolimus, 33 ER tacrolimus) followed for 5 years. NODAT was defined according to standard criteria. In vitro dissolution testing was performed at pH values of 1.2, 4.5, and 6.8, with sampling at 15, 30, 60, 90, 120, and 360 min. **Results**: The obtained results do not indicate differences regarding the incidence of diabetes mellitus in patients treated with the two forms of tacrolimus. The determined NODAT incidence was 42.4% (ER tacrolimus) vs. 39.4% (IR tacrolimus), *p* = 0.802, and ER tacrolimus showed slower release without significant pH-dependent variations. **Conclusions**: No significant differences in NODAT incidence were identified between formulations. The release–clinical outcome correlation requires validation in larger multicenter studies. These results contribute to the evidence base for tacrolimus formulation selection in renal transplant patients and other associated pathologies.

## 1. Introduction

Renal transplantation represents the most efficient renal replacement therapy in patients with end-stage kidney disease (ESKD). Sustaining graft viability depends on the efficacy of immunosuppressive therapy in mitigating the immune system’s response against non-self antigens. Calcineurin inhibitors, including cyclosporine and tacrolimus (TAC), are considered first-line immunosuppressive agents in current therapeutic protocols. Beyond proven efficiency, TAC has been shown to exert detrimental effects on pancreatic β-cell function and viability, favoring the development of insulin resistance, obesity, and diabetes mellitus [1]. Diabetes mellitus is a post-transplant complication that affects a proportion of up to 30% of these patients, considerably increasing the risk for cardiovascular diseases and death [2].

The TAC pharmacokinetic profile depends on the therapeutic formulation. Immediate-release forms (IR-TAC) with twice-daily dosing regimens are absorbed faster, at the level of the proximal small intestine, while delayed-release forms (ER-TAC) administered once daily are absorbed more slowly, at the level of the distal small intestine, thereby ensuring a more stable blood concentration [3]. The different bioavailability of the two TAC formulations also requires different dosing of the active substance, with up to 30% lower in immediate-release pharmaceutical forms [4,5].

To prevent toxicity and maintain allograft function, various monitoring protocols aim to keep tacrolimus (TAC) levels between 7 and 12 ng/mL during the first month post-transplant. This approach helps reduce the risk of acute graft rejection, with the dosage being individualized over time for each patient based on their blood TAC levels. This aspect is crucial for minimizing tacrolimus (TAC) toxicity, particularly because small changes in dosage can have significant adverse effects on both the graft and the patient. Thus, reducing the TAC level between 4 and 7 ng/mL increases the risk of graft rejection by 86%. On the other hand, TAC concentrations between 5.35 and 7.15 ng/mL significantly prevent the risks of developing infections [6]. Thus, the boundary between toxicity and immunological efficacy is extremely narrow. The serum level of TAC depends on its metabolism rate, which is influenced by multiple factors, including genetic variability. Scientific studies have indicated that patients with the CYP3A5 gene have a clearance rate 1.48 times higher compared to patients who do not express it [7]. To minimize the numerous adverse effects associated with immunosuppressive therapy and to optimize renal graft survival, personalized therapeutic protocols have been developed.

**Figure 1:**
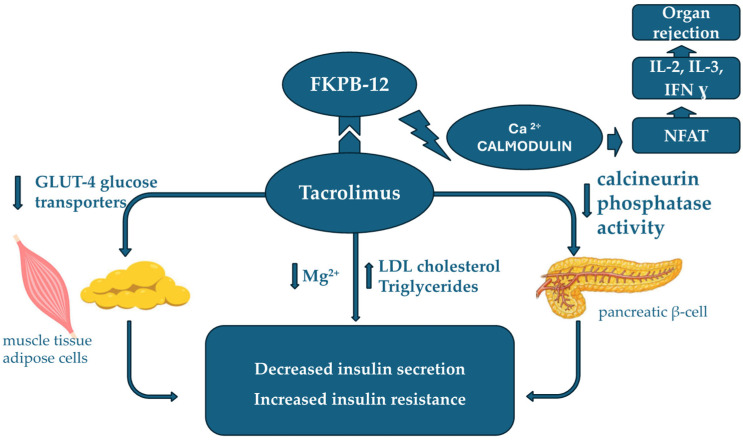
Mechanism of action of TAC. The involvement of tacrolimus in the development of post-transplant diabetes mellitus. Legend: NFAT—nuclear factor of activated lymphocytes; IL—interleukin; FKBP-12—FK binding protein; NFAT—nuclear factor of activated T-cells; IL—interleukins; IFN–interferon.

TAC is the most used drug with immunosuppressive action in renal transplant patients, and the highly variable pharmacokinetics requires the most efficient dosing possible. Variability even in the same patient is primarily related to cytochrome P450 polymorphisms, which play a central role in TAC metabolism. Tacrolimus (TAC) bioavailability is also influenced by several non-genetic factors, including diet, gastrointestinal motility, concomitant medications (e.g., corticosteroids and calcium channel blockers), hemoglobin levels, and patient-specific characteristics such as sex, age, and body weight [8,9]. The mechanism of action of TAC is presented in Figure 1. Inhibition of the calcium–calmodulin complex by TAC coupled with FK binding protein (FKBP) will result in blocking the nuclear factor of activated lymphocytes (NFAT). This inhibition prevents the transcription of genes involved in the synthesis of pro-inflammatory cytokines, interleukins IL2, IL3, and IFN gamma, thereby reducing the risk of immune rejection following organ transplantation.

Extended-release formulations of tacrolimus (TAC) have been developed to improve patient compliance and reduce fluctuations in blood concentrations associated with the rapid release of the active substance from immediate-release capsules. These formulations enable the controlled, sustained release of TAC at a defined rate over a prolonged period, thereby maintaining more stable serum levels and potentially reducing dose-dependent adverse effects. This challenge is largely attributed to tacrolimus’s (TAC) low water solubility and limited bioavailability. To address this, a once-daily formulation was developed to enhance TAC absorption by creating a solid dispersion of the drug through a physical process known as controlled agglomeration [10].

The incidence of new-onset diabetes after transplantation (NODAT) has been extensively investigated by numerous research groups, with findings indicating only minor differences between immediate-release (IR-TAC) and extended-release (ER-TAC) formulations. A study conducted by Silva et al. comparing the two formulations reported a NODAT incidence of 41.1% in patients treated with Astagraf XL (Astellas, once daily) and 33.6% in those receiving Prograf (Astellas, twice daily), with both formulations demonstrating comparable safety and efficacy profiles [11]. Another study conducted by Torres et al. comparing two IR-TAC and ER-TAC formulations obtained similar values of NODAT incidence: 18.5% for the immediate-release formulation and 24% for the extended-release formulation [12].

**Figure 2:**
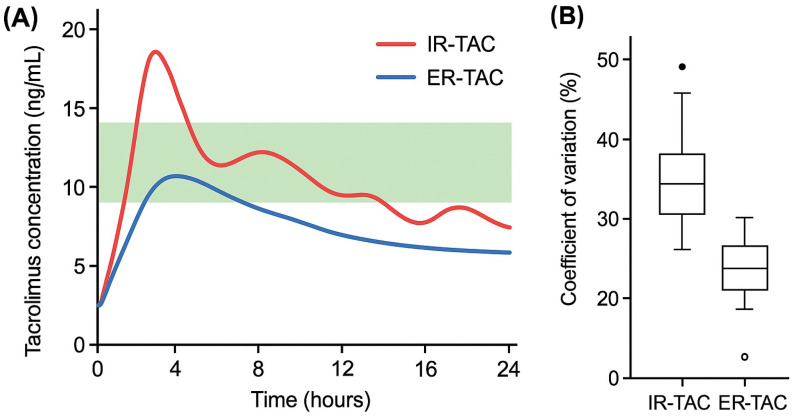
Pharmacokinetic profiles of immediate-release (IR) and extended-release (ER) tacrolimus formulations. (**A**) Representative 24 h serum concentration profiles showing twice-daily immediate-release (IR-TAC, red line) versus once-daily extended-release (ER-TAC, blue line) tacrolimus. The green shaded area represents the optimal therapeutic range (5–15 ng/mL). IR-TAC shows characteristic biphasic peaks with greater fluctuations, while ER-TAC demonstrates more sustained levels with reduced variability. (**B**) Intra-patient variability comparison showing coefficient of variation (CV%) for trough levels. ER-TAC formulation demonstrates significantly lower variability compared to IR-TAC.

Since the TAC level in blood depends on a multitude of factors that are related to the patient as well as the administered dose, TAC blood dosing is essential for dose individualization. To determine the effective tacrolimus (TAC) dose—regardless of the formulation—frequent monitoring of TAC blood levels is necessary, particularly during the immunologically unstable period immediately following renal transplantation. Several studies have demonstrated the safety and efficacy of both immediate-release (IR-TAC) and extended-release (ER-TAC) formulations at 12 months, 2 years, and 4 years post-transplantation. The findings of these studies support the hypothesis that both formulations are safe and effective, with only minor differences in clinical outcomes. However, the extended-release tacrolimus (ER-TAC) formulation demonstrates superior absorption and bioavailability compared to the immediate-release formulation (IR-TAC) [13,14,15]. However, the selection of the tacrolimus dose, dosing regimen, and pharmaceutical formulation remains the responsibility of the attending physician and is adjusted based on the individual patient’s serum TAC levels. These aspects are presented schematically in Figure 2.

Newer alternatives to TAC (belatacept) reduce the risk of post-transplant diabetes mellitus but present a higher risk of acute graft rejection [16].

The main objective of this pilot study was to compare the incidence of NODAT between renal transplant patients treated with IR-TAC versus ER-TAC formulations over a five-year follow-up period. Secondary objectives included characterizing the in vitro release profiles of formulations across different pH values and exploring potential correlations between these release characteristics and observed clinical outcomes. The results of this pilot study will inform the design of prospective multicenter studies and will contribute to optimizing TAC formulation selection in clinical practice.

## 2. Results

### 2.1. Dissolution Test and Tacrolimus Dosing

In order to determine the dissolution time at different pH values, we conducted studies on the pharmaceutical forms included in the therapeutic regimen of patients included in the study. The industrial products on which the analyses were performed are included in Table 1.

TAC monohydrate is obtained through a fermentation process in the presence of bacteria, and after several processes, the crude product is obtained, which is subsequently purified and crystallized. Purity, crystal size, and shape differ from one manufacturer to another and are important characteristics that influence TAC bioavailability [17]. Particle size reduction favors solubility, but in the case of TAC, this is not sufficient because it has very low water solubility and implicitly reduced bioavailability after oral administration. The association of TAC with hypromellose ensures increased TAC solubility in water as a result of transformation from the crystalline to amorphous form, but TAC stability is also achieved through the formation of intermolecular hydrogen bonds that prevent reversion to the crystalline form [18]. Tsunashima et al. demonstrated the importance of excipients used in Advagraf preparation for prolonged TAC release, with formulations without hypromellose having a reduced degree of supersaturation (after 4 h) compared to formulations containing hypromellose (over 24 h) [19]. The quantity and type of hypromellose in the formulation influence the release characteristics of the active substance; formulations in which hypromellose has a higher molecular weight have a high water retention capacity and reduced erosion of the formed gel, which means delayed release of the active substance [20]. The difference between formulations consists in the nature of excipients and implicitly in the release mode of the active principle. Regarding the nature of excipients, we note a single difference, namely the presence of ethylcellulose in the composition of extended-release capsules and at the same time the replacement of this with croscarmellose sodium in immediate-release capsules.

Ethylcellulose is a cellulose derivative insoluble in water but permeable to water regardless of pH. It forms an insoluble film that does not change when crossing the gastrointestinal tract, acting as a permeable membrane for digestive fluids. The active substance is released by diffusion in a controlled manner. In combination with hypromellose, a gastrosoluble film is obtained that ensures the extended release of TAC from the pharmaceutical form [21].

Croscarmellose is cross-linked sodium carboxymethylcellulose with a high absorption and swelling capacity in a short time of only a few seconds. These properties give it the role of a disintegrating agent, being practically the most efficient superdisaggregate agent.

Magnesium stearate has lubricating action, and lactose monohydrate is a diluent. Hypromellose is a water-soluble cellulose derivative dependent on pH and controls the release of the active substance, being soluble in acidic medium.

The kinetics of the TAC release process from pharmaceutical forms depends on the rate of hydration, imbibition, dissolution, diffusion, and erosion of the gel layer that forms upon contact with water and digestive fluids. The active substance is progressively released through the gel barrier around it along with the destructuring of the matrix.

**Figure 3:**
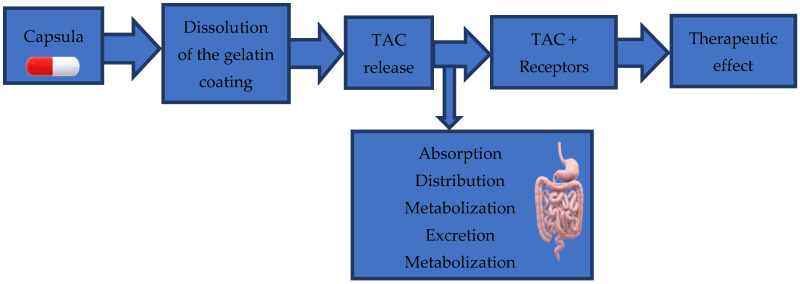
Scheme of processes that occur after administration of TAC capsule.

The processes that occur after administration of the TAC capsule and until obtaining the therapeutic effect are presented in Figure 3.

In order to increase bioavailability, amorphous solid dispersion formulations have been developed, obtained by transforming the crystalline form into an amorphous form [22,23]. To combat the thermodynamic instability of the amorphous form, polymeric compounds are used as excipients, which, through the formation of intermolecular bonds, not only provide stability but also increase solubility and implicitly the oral bioavailability of TAC. Among all polymers used as excipients, hypromellose has proven to be the most efficient in the case of immediate and extended-release TAC and in combination with ethylcellulose in the case of extended-release forms [18,24,25].

In the case of immediate-release pharmaceutical forms with twice-daily administration (Prograf and tacrolimus), faster release is observed regardless of pH value compared to the extended-release preparation (Advagraf). This aspect is important because faster TAC release from the pharmaceutical form is associated with reaching serum levels faster than after Advagraf administration. Depending on the dose administered to each patient, the serum TAC level differs, but the TAC release percentage below 50% from Advagraf indicates a lower serum TAC level compared to immediate-release forms, where there are variations in the serum TAC level as a result of faster release from the pharmaceutical form. A high serum TAC concentration in the long term may be associated with dose-dependent adverse effects. A minimum effective TAC concentration reduces the incidence of dose-dependent adverse effects. Therefore, the pharmaceutical form formulation directly influences the time and rate of active principle release from the capsule and favors the occurrence of dose-dependent adverse effects. The results obtained following the dissolution test indicate slower TAC release from extended-release capsules, but pH does not significantly influence the active substance release rate from the pharmaceutical form neither in the case of immediate-release capsules nor those with delayed release. Table 2 presents the results obtained following the dissolution test.

TAC metabolism is performed with the help of the CYP3A4 enzyme, and the activity of this enzyme is inhibited by calcium channel blockers, antifungals, and certain antibiotics but also foods, such as grapefruit juice. An inducing action of this enzyme is performed by rifampicin and anticonvulsants. Administration of medications and/or foods before or immediately after TAC administration can modify its absorption as a result of modifying the pH of the dissolution medium or the action exerted on the TAC metabolizing enzyme. Changes that influence TAC absorption generate variations in its serum level with implications regarding therapeutic effect and possible adverse reactions. In this regard, we performed determinations of TAC capsule dissolution at different pH values: pH 1.2 (artificial gastric juice), pH 4.5, and 6.8 (artificial intestinal juice).

### 2.2. Clinical Impact Study

The mean age of the patients included in our study was 50.273 ± 10.14 years old, with no significant differences between the two groups, immediate-release or extended-release tacrolimus treatment (50.121 ± 11.683 vs. 50.424 ± 8.511, *p* = p.905). The majority were male (53%), in a similar proportion between groups (*p* = 0.62) and with a mean transplant vintage of 14 ± 6.919 (*p* = 0.673). The patients were generally overweight, with a BMI of 26.285 ± 3.503, slightly increased in the IR-TAC group (26.709 ± 3.013 vs. 25.861 ± 3.934, *p* = 0.329). Most of the patients in both groups received renal allograft from deceased donors (72.72% vs. 84.84%, *p* = 0.366), and the documented acute rejection episodes appeared at an equal rate (12.1%). We found a significant difference in corticotherapy usage in favor of the IR-TAC group (100% vs. 81.81%, *p* = 0.024), while the mean tacrolimus dose, although higher in the IR-TAC group, was not statistically different (4.227 ± 1.663 vs. 3.727 ± 1.596, *p* = 0.217). We did not document differences in allograft function between groups, with a mean eGFR of 61.98 ± 28.045 and a creatinine value of 1.468 ± 0.686. The patient groups were also homogenous in terms of laboratory parameters, with no statistically significant differences in the serum level of glucose, HbA1c, hs-CRP as inflammatory biomarker, uric acid, calcium, magnesium, cholesterol, triglycerides, and urinary proteins (Table 3). NODAT was diagnosed in similar proportion, without a significant statistical relevance between groups (39.39% vs. 42.42%, *p* = 1). The patient characteristics are presented in Table 3.

Patients with NODAT were younger (49.111 ± 8.541 vs. 51.077 ± 11.155, *p* = 0.583), with a higher BMI (27.619 ± 3.459 vs. 25.362 ± 3.266, *p* = 0.008), and with a lower transplant vintage (10.519 ± 3.469 vs. 16.410 ± 7.687, *p* = 0.07). The documented acute rejection episodes were recorded in similar proportion as well as the corticotherapy usage. The mean dose of tacrolimus was lower in NODAT group (3.593 ± 1.494 vs. 4.244 ± 1.697, *p* = 0.147). In terms of laboratory findings, diabetic patients, in addition to elevated blood glucose and glycated hemoglobin levels, showed significantly higher triglyceride (184.111 ± 42.95 vs. 136.632 ± 45.563, *p* < 0.001) and proteinuria levels (0.883 ± 0.73 vs. 0.498 ± 0.876, *p* < 0.001), along with reduced serum magnesium concentrations (1.618 ± 0.148 vs. 1.752 ± 0.143, *p* < 0.001) (Table 4).

When comparing the patients with NODAT with regard to tacrolimus formulation usage, the group treated with ER-TAC is older (51.786 ± 5.563 vs. 46.231 ± 10.35, *p* = 0.103), with a higher BMI (28.35 ± 3.6 vs. 26.831 ± 3.255, *p* = 0.174), a higher proportion of deceased donor allograft (50% vs. 23%), and with a significantly lower transplant vintage (8.429 ± 2.344 vs. 13.077 ± 3.095, *p* < 0.001). The allograft function did not differ significantly in the patients with NODAT receiving the ER-TAC vs. IR-TAC formulation (eGFR 56 ± 24.019 vs. 64.769 ± 29.329, *p* = 0,512), although the proteinuria was considerably higher in ER-TAC NODAT patients. Hs CRP as an inflammation biomarker was slightly higher in the patients using ER-TAC (6,621 ± 5.535 vs. 5.054 ± 4.893, *p* = 0.243), as were triglycerides (196.429 ± 26.795 vs. 170.846 ± 53.412, *p* = 0.174) and cholesterol (207.636 ± 61.74 vs. 193.375 ± 32.53, *p* = 0.431) (Table 5).

**Figure 4:**
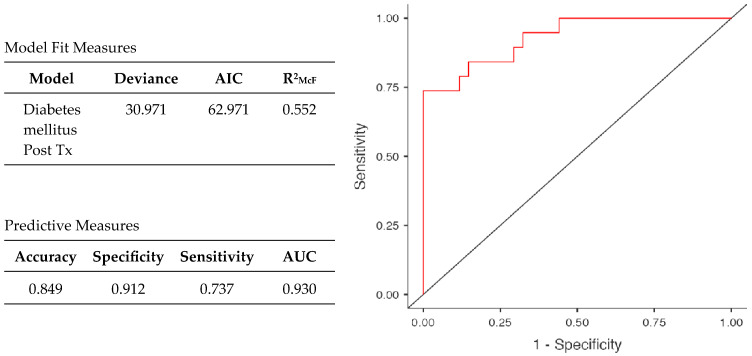
Multivariate logistic model characteristics.

The logistic multivariate analysis included the main parameters with potential predictive value for the development of NODAT: age, gender, type of donator, transplant vintage, incidence of acute rejection, type of tacrolimus formulation, corticotherapy usage, eGFR, BMI, CRP as inflammatory biomarker, cholesterol, triglycerides, magnesium, uric acid, and proteinuria. This model provided the best goodness of fit measure (AIC = 62.971, McFadden’s R^2^ = 0.552) and valuable predicted power (AUC of 0.930) (Table 6, Figure 4).

**Figure 5:**
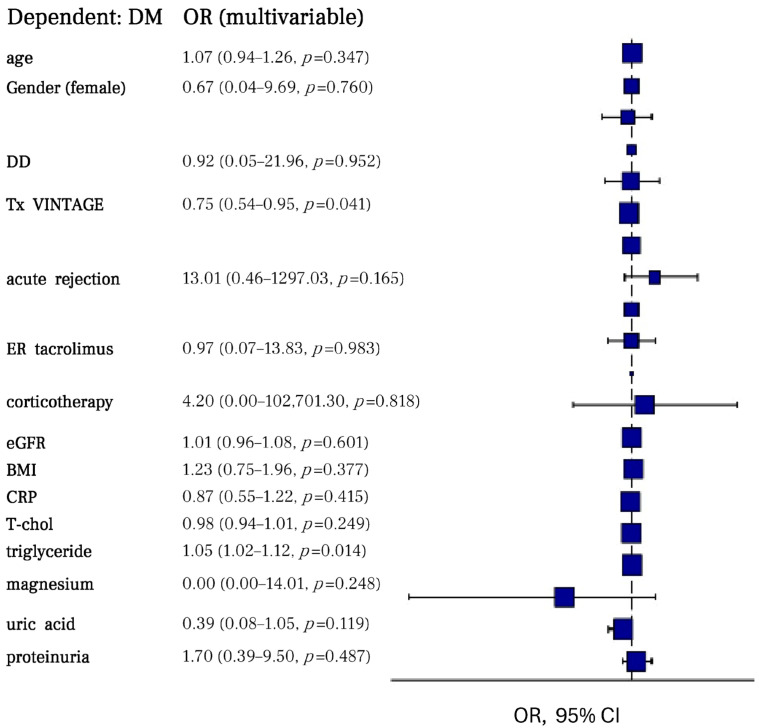
Odds Ratio plot for NODAT predictors.

The Odds Ratio (multivariate) plot revealed a strong correlation between NODAT and acute rejections (13.01 (0.46–1297.03, *p* = 0.165), associated corticotherapy (4.20 (0.00–102,701.30, *p* = 0.818)), decreased magnesium level (0.00 (0.00–14.01, *p* = 0.248)), and increased proteinuria (1.70 (0.39–9.50, *p* = 0.487)). However, none of aforementioned parameters reached statistical significance (*p* > 0.05) (Table 7, Figure 5).

## 3. Discussion

### 3.1. NODAT After Renal Transplantation

The development of new-onset diabetes after transplantation (NODAT) is multifactorial, influenced by modifiable risk factors such as immunosuppressive therapy, obesity, metabolic syndrome, proteinuria, hypomagnesemia, and certain infections, as well as by non-modifiable factors including age, sex, donor organ characteristics, pre-existing glucose intolerance, and a history of corticosteroid therapy.

The study of the adverse effects of immunosuppressive drugs remains a constant concern in transplant patients and requires careful dose monitoring to balance drug toxicity with the prevention of graft rejection. Corticosteroid therapy and calcineurin inhibitors are essential components of the immunosuppressive regimen following renal transplantation. While numerous studies have demonstrated the beneficial effects of corticosteroid therapy in preventing graft rejection, long-term administration is associated with multiple adverse effects—most notably, a 42% increased risk of NODAT. This risk is linked to mechanisms such as enhanced peripheral insulin resistance and pancreatic beta-cell apoptosis, particularly at doses exceeding 10 mg [26,27]. Beyond insulin resistance and diabetes mellitus, corticosteroids are additionally associated with hyperlipidemia, obesity, and elevated cardiovascular risk [28].

Tacrolimus (TAC) exhibits diabetogenic properties, which are more pronounced with concomitant administration of high-dose glucocorticoids. NODAT represents a significant adverse effect of tacrolimus (TAC) therapy, with reported incidence rates reaching up to 53% in some studies [29], Glucose intolerance induced by calcineurin inhibitors is attributed to decreased insulin secretion, increased insulin resistance, and direct toxicity to pancreatic β-cells—the binding site of the tacrolimus-specific protein FKBP-12. Tacrolimus-induced toxicity at the pancreatic β-cell level leads to reduced insulin production by interfering with calcineurin phosphatase activity within pancreatic tissue [30,31]. Calcineurin plays a crucial role in maintaining proper pancreatic β-cell function. Calcineurin inhibitors promote hyperglycemia by downregulating genes involved in enhancing insulin sensitivity in muscle tissue and by reducing GLUT-4 glucose transporters in muscle and adipose cells. Furthermore, the calcineurin-NFAT signaling pathway contributes to the development of insulin resistance, which is a key mechanism in NODAT pathogenesis. The degree of hyperglycemia exhibits significant inter-patient variability and is directly influenced by TAC dosage C [32]. Moreover, TAC administration generates increased levels of low-density lipoprotein cholesterol and triglycerides and a decrease in high-density lipoprotein cholesterol [33]. NODAT is observed more frequently in patients with hypomagnesemia [34]. Both magnesium and tacrolimus (TAC) influence sodium and potassium ion homeostasis, thereby contributing to renal tubular dysfunction and associated nephrotoxic effects [35,36]. The toxic effects of tacrolimus (TAC) are not only dose-dependent but are also influenced by its metabolic processing within the body.

In our patients, among whom the incidence of diabetes was over 40%, tacrolimus-based therapy represented the main component of the immunosuppressive regimen. Both immediate-release and extended-release formulations were used to an equal extent, significantly surpassing the use of cyclosporine. In a significant proportion of these patients, we observed the onset of NODAT within the first two years post-transplant. However, in our patient cohort, we were unable to demonstrate a clear correlation between post-transplant diabetes and tacrolimus administration, as the multivariate analysis indicated that the development of NODAT involved a combination of contributing factors.

Obesity represents an important risk factor for NODAT, which is explained by the involvement of adipose tissue in IL-6 synthesis, leading to increased insulin resistance. As adipose tissue increases, levels of TNF-α also rise, which alters glucose receptor phosphorylation and contributes to the development of insulin resistance [37]. Additionally, adipose tissue induces decreased adiponectin secretion, which favors NODAT development [38]. In this regard, Roland et al. conducted a study on 857 renal transplant patients and demonstrated that BMI represents a risk factor in NODAT development, with incidence varying from 1.5% for BMI under 25 to 5.6% for BMI over 30 [39]. A study of 640 renal transplant patients without diabetes showed that 34.4% of those with metabolic syndrome (characterized by high blood pressure, elevated blood glucose, cholesterol, triglycerides, etc.) developed NODAT compared to 27.4% of patients without metabolic syndrome. Additionally, the percentage of patients who developed NODAT increased with the number of metabolic syndrome components, ranging from 24.2% in those with one component to 73.7% in those with five components [40].

In our cohort, the vast majority of patients were overweight, and among those who developed diabetes, the weight gain was statistically significantly higher compared to non-diabetic patients. Additionally, the dyslipidemia profile of patients with NODAT was distinct, characterized by significantly higher triglyceride levels as well as elevated total cholesterol and LDL cholesterol levels.

Several other factors may also be linked to the onset of NODAT. Van Laecke et al. investigated the relationship between NODAT incidence and magnesium levels, age, BMI, and triglyceride values in 390 renal transplant patients. The results of this study indicate that hypomagnesemia and treatment with calcineurin inhibitors are associated with NODAT incidence as a result of blocking the renal magnesium transporter, which favors urinary magnesium loss and hypomagnesemia [41]. Extensive studies involving 828 patients have shown that low-grade proteinuria (<1 g/day) and very-low-grade proteinuria (<0.3 g/day) are independent risk factors for NODAT, regardless of age, sex, BMI, baseline fasting glucose, blood pressure, and use of antihypertensive medications [42]. Similar to the aforementioned studies, in our patients, NODAT was significantly correlated with hypomagnesemia and proteinuria, without statistically significant differences regarding graft function or the post-transplant period.

Regarding the age of renal transplant patients, studies indicate that those over 45 years are more predisposed to NODAT. Additionally, the incidence of NODAT is influenced by donor type, with patients receiving organs from deceased donors being more prone to develop NODAT compared to those who received transplants from living donors. This difference is related to the fact that immunosuppressive therapy tends to be more aggressive in cases involving deceased donors, and prolonged ischemia may cause certain lesions [43]. In our study cohort, with an average age of approximately 50 years, the grafts were predominantly obtained from donors with brain death, and corticosteroid therapy was almost universally included in the post-transplant treatment regimens.

### 3.2. Tacrolimus Metabolism and Side Effects in Renal Transplantation

Tacrolimus, along with cyclosporine, represents a key component of immunosuppressive therapy and requires careful dose management due to its nephrotoxic, neurotoxic, cardiotoxic, and metabolic side effects. Although both cyclosporine and tacrolimus (TAC) share a similar mechanism of action by inhibiting calcineurin, TAC exerts therapeutic effects at concentrations up to 100 times lower than cyclosporine, thereby reducing the risk of adverse effects associated with this class of immunosuppressants [44,45].

In general, younger individuals metabolize tacrolimus (TAC) faster than elderly individuals. Genetic factors, such as variations in the POR and ABCB1 genes, affect TAC metabolism and may contribute to the development of diabetes mellitus [46]. On the other hand, the involvement of cytochrome P450 enzymes CYP3A4 and CYP3A5 in tacrolimus (TAC) metabolism contributes to drug–drug interactions by altering its pharmacokinetics [47,48]. Tacrolimus (TAC) metabolism occurs primarily in the liver, involving demethylation and hydroxylation processes that produce secondary and tertiary metabolites, which extend its half-life from 12 to 15 h. Tacrolimus (TAC) metabolism is associated with P-glycoprotein activity, which facilitates the transport of TAC metabolites from the liver to the intestine, the primary site of absorption [49].

Given the numerous adverse effects associated with tacrolimus (TAC) administration, personalized therapy is essential to optimize therapeutic efficacy while minimizing toxicity. The optimal tacrolimus (TAC) dose is closely related to its clearance and is influenced by CYP3A4 gene polymorphisms, which contribute to significant interindividual variability. Consequently, TAC dose adjustments ranging from 20% to 60% may be required, depending on the individual’s metabolism rate [50,51]. Although genetic variability significantly influences tacrolimus (TAC) metabolism and the determination of the optimal dose, the high cost and limited accessibility of genetic testing pose substantial barriers to incorporating this approach in personalized TAC dosing.

To minimize adverse effects resulting from excessively high tacrolimus (TAC) doses, some researchers have calculated the ratio of the minimum TAC concentration required for therapeutic effect to the corresponding dose of immediate-release TAC (C/D ratio). A high C/D ratio indicates a slow metabolism rate, whereas a low ratio reflects rapid metabolism [52]. To achieve stable blood levels of tacrolimus (TAC), extended-release formulations have been developed, exhibiting pharmacokinetic profiles distinct from those of immediate-release formulations [53]. Several studies measuring tacrolimus (TAC) blood levels after administration of immediate-release and extended-release formulations support the hypothesis that the C/D ratio is higher following extended-release TAC administration. Unlike immediate-release forms, which are primarily absorbed in the proximal small intestine, extended-release formulations undergo absorption throughout the distal small intestine and large intestine, resulting in improved bioavailability [54,55,56,57,58]. Extensive studies involving over 1000 patients in France have aimed to establish the optimal tacrolimus (TAC) dosing for both immediate- and extended-release formulations to achieve effective blood concentrations while minimizing adverse effects. TAC blood levels were measured at 3, 6, and 12 months post-transplantation, demonstrating stabilization of the C/D ratio by 12 months, coinciding with graft function stabilization [59]. Administration of tacrolimus (TAC) in extended-release formulations is associated with improved renal function, with patients demonstrating higher estimated glomerular filtration rate (eGFR) values 12 months after renal transplantation [52].

Another important factor significantly affecting tacrolimus (TAC) metabolism and absorption is drug and food interactions, which can alter cytochrome P450 enzyme activity and consequently increase or decrease TAC blood levels [49].

Immediate-release formulations of tacrolimus (TAC) do not maintain stable plasma levels over extended periods, necessitating twice-daily dosing. In contrast, extended-release formulations provide sustained TAC release, helping to prevent dose-dependent adverse reactions [60]. Achieving the desired therapeutic effect largely depends on the release characteristics of the pharmaceutical formulation. In this context, the type and concentration of the disintegrant play a critical role in controlling TAC release [61,62].

Ethylcellulose is an ethyl ether of cellulose frequently used in the pharmaceutical industry, being insoluble in the body regardless of pH and permeable to water, but in the presence of gastric juice, it increases its volume [63]. These characteristics of ethylcellulose have led to its use in various pharmaceutical formulations with modified release of the active substance, thus ensuring a constant level of the active principle for a longer period of time compared to immediate-release pharmaceutical forms. This aspect is important also from the perspective of reducing the frequency of dose administration and pharmacotherapeutic efficiency [64]. Through the use of solid dispersion techniques, ethylcellulose has been used as a matrix for water-soluble or insoluble active substances, allowing their extended release [65]. Regarding TAC, ethylcellulose ensures its delayed release from the industrial preparation Advagraf, with approximately 60% being released after 8 h regardless of the pH value of the dissolution medium [66]. Also, using the solvent evaporation method and ethylcellulose and hypromellose as polymers, Tsunashima et al. obtained a delayed-release formulation with TAC as the active principle, also managing to improve its solubility [19]. The diffusion of the active principle through the polymeric layer depends on its solubility. Usually, to ensure the delayed release of the active principle from the pharmaceutical form, a mixture of polymers is used. As a result of the hydrophobic character of ethylcellulose, it reduces water penetration into the formed matrix and thus slows down the release of the active principle [67].

In order to increase treatment adherence, modified-release preparations have been formulated, with once-daily administration and lower serum levels compared to immediate-release preparations with twice-daily administration [68]. Results obtained by Rostaing et al., conducted on 543 patients at 2 years after renal transplantation, indicate differences regarding bioavailability between Prograf and Advagraf, with an average dose increase of 8% being necessary when switching from Prograf to Advagraf [15]. Each patient’s doses must be calculated based on the minimum concentration correlated with the area under the curve, even though these values are variable [69]. An extremely important factor in TAC bioavailability is the presence of CYP3A enzymes at the liver and intestinal wall level. After oral administration, a large part of TAC is metabolized at the intestinal level [70]. Berggren et al. demonstrated that CYP3A activity is not uniform throughout the intestinal surface, being higher in the upper part of the small intestine and decreasing toward the colon [71]. This aspect is very important regarding TAC bioavailability because IR-TAC is absorbed at the proximal small intestine level while ER-TAC is absorbed at the distal small intestine level. In a study conducted on predominantly Caucasian patients, a significant influence of CYP3A5 on serum TAC levels was found. The minimum TAC concentration decreased significantly in patients who do not express CYP3A5, while in those who express it, it remained constant when switching from Prograf to Advagraf [72]. Although there are few exceptions, most clinical studies indicate that in renal transplant patients with CYP3A5, serum TAC levels increase following the switch from Prograf to Advagraf compared to patients who do not express CYP3A5 [73]. Therefore, pharmacokinetic variability influences the pharmacokinetics of TAC formulations, in the sense that there is a greater change in minimum TAC levels in patients who express CYP3A5 compared to those who do not express it.

A novel aspect of this study is the evaluation of the dissolution time of tacrolimus (TAC) pharmaceutical formulations used in the immunosuppressive therapy of renal transplant patients at our center. Tacrolimus (TAC) dosing from pharmaceutical formulations was assessed at various pH levels to investigate potential differences related to the impact of drugs or foods that alter the physiological pH of the digestive tract, thereby affecting TAC bioavailability and the risk of dose-dependent adverse effects. Although only minor differences were observed in the tacrolimus (TAC) release rate from immediate-release formulations, even small variations in the amount of active substance released can significantly impact serum TAC levels and, consequently, the risk of adverse effects during prolonged treatment. The results for tacrolimus (TAC) release from delayed-release capsules indicate a prolonged release of the active substance, which helps minimize fluctuations in serum TAC levels and allows for reduced dosing frequency.

Although this study has limitations due to its retrospective design and the relatively small number of patients treated with different tacrolimus (TAC) formulations, the findings may complement data from other centers to help refine therapeutic protocols for renal transplant patients. This is particularly important given the significant inter- and intra-patient variability in TAC pharmacokinetics. Furthermore, despite the advantages of prolonged-release formulations in maintaining stable serum TAC levels, our results indicate no significant difference in the incidence of new-onset diabetes after transplantation (NODAT) as a potential adverse effect of extended TAC therapy.

Given the results of numerous studies demonstrating a clear association between calcineurin inhibitor therapy and the high incidence of post-transplant diabetes mellitus (NODAT) in transplant recipients, a possible explanation for our findings is that, despite the controlled release of tacrolimus (TAC) from the pharmaceutical formulation, other factors significantly influence serum TAC levels and consequently the development of dose-dependent adverse effects. To reduce the incidence of NODAT, it is important to consider the impact of the formulation on serum TAC concentrations. Additionally, the minor differences in NODAT incidence observed between patients treated with Advagraf and those receiving immediate-release TAC formulations (Prograf and Tacrolimus) may also be attributable to genetic factors affecting TAC metabolism.

In this context, studies investigating the transition to the colon-controlled release formulation, Envarsus, which utilizes MeltDose technology to incorporate tacrolimus (TAC) with enhanced bioavailability through molecular-level particle size reduction and the formation of a ‘solid solution’, would be particularly valuable.

The lower expression of CYP3A5 in the colon may allow for an average tacrolimus (TAC) dose reduction of approximately 36% with Envarsus compared to Advagraf, which is also a once-daily controlled-release formulation. This contributes to maintaining stable TAC levels even in patients exhibiting slow absorption and rapid metabolism [74].

From a pharmaceutical perspective, in vitro studies confirmed expectations, with immediate-release tacrolimus (IR-TAC) exhibiting faster and more immediate release of the active compound compared to extended-release TAC (ER-TAC). However, the influence of TAC release characteristics on the incidence of new-onset diabetes after transplantation (NODAT) appears to be more significantly affected by patient-specific factors such as age, donor type, cardiovascular comorbidities, and genetic determinants of drug metabolism. Despite the absence of significant differences in NODAT incidence between formulations, the sustained-release properties of extended-release tacrolimus (ER-TAC) may provide benefits in dosing convenience and potentially other clinical outcomes not evaluated in this study. The once-daily dosing regimen of ER formulations could enhance patient adherence, a critical factor for long-term graft survival.

While large prospective studies remain the gold standard for comparative effectiveness evaluation, our study provides the first direct comparison of these three specific formulations within the Romanian healthcare context, contributing real-world evidence that complements controlled study results.

## 4. Materials and Methods

We conducted a retrospective, observational, single-center pilot study between January 2020 and December 2024 on 66 patients with renal transplantation under the care of Bihor County Emergency Clinical Hospital, Nephrology Department. This study was designed to compare the incidence of NODAT between IR-TAC and ER-TAC formulations in renal transplant recipients while correlating clinical outcomes with in vitro drug release characteristics. This study complies with the Declaration of Helsinki and Good Clinical Practice guidelines and received the approval of the Ethics Committee of the Bihor County Clinical Emergency Hospital (approval number 5078/13.02.2025). Given the retrospective nature of this study, the requirement for individual informed consent was waived by the ethics committee, with patient confidentiality maintained through data anonymization.

### 4.1. Study Design

The patients were followed for 5 years, between January 2020 and December 2024.

The inclusion criteria were (a) age over 18 years old; (b) a history of more than 5 years since kidney transplantation at the time of follow-up index; (c) absence of diabetes prior to the transplant; (d) immunosuppressive regimen including tacrolimus as CNI.

The exclusion criteria were (a) failure to meet the previously mentioned time criteria; (b) cyclosporine treatment; (c) incomplete data regarding the medical history; (d) loss of follow-up due to death or transition to other forms of renal replacement therapy; (e) combined organ transplant.

After completing the selection algorithm, 66 kidney transplant patients undergoing treatment with tacrolimus were included in our study: 33 receiving the immediate-release formulation, namely, Prograf (Astellas and Sandoz), and 33 the extended-release formulation (Advagraf, Astellas). The allocation of the medication was decided by the Renal Transplant Institute, which was also responsible for initiating any changes in the therapy regimen or dose adjustment based on tacrolimus blood level.

**Figure 6:**
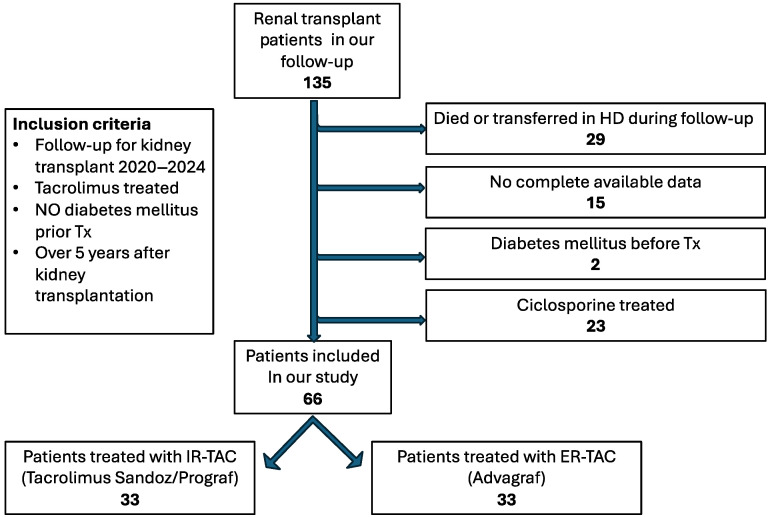
Flowchart for patient selection.

The selection algorithm is illustrated in Figure 6.

### 4.2. Study Objectives

Based on the particularities of the tacrolimus formulation metabolism, the aim of this study was (a) to evaluate the incidence of diabetes mellitus in the patients receiving tacrolimus and (b) to demonstrate the involvement of the tacrolimus formulations in the development of diabetes mellitus after transplantation

### 4.3. Data Collection

The data were collected from the GP files (2020–2024) and specialized outpatient clinical documentation and included the following:

(a) Demographic data: age, age after transplantation, and gender;
(b) Data related to transplant procedure: type of donator and acute rejection episodes;
(c) Biometric data: body mass index (BMI);
(d) Treatment data: type of tacrolimus formulation, median tacrolimus dosage, the use of corticotherapy, and type of antidiabetic treatment, e.g., insulin, oral antidiabetics, diet;
(e) Laboratory analyses: creatinine (0.7–1.3 mg/dL), eGFR, fasting glucose (74.00–100.00 mg/dL), Hb A1c (4–5.7%), hs-CRP (0–5 mg/dL), AST (11–34 U/L), triglycerides (0–150.00 mg/dL), total cholesterol (0–200.00 mg/dL) and LDL cholesterol (10.00–100.00 mg/dL), calcium (8.5–10.2), magnesium (1.6–2.6 mg/dL), uric acid (3.7–7.7 mg/dL), and proteinuria; the glomerular filtration rate (eGFR) was estimated by applying the CKD-EPI 2021 formula according to the KDIGO 2024 recommendation.

HbA1c level was assessed using an Abbott ALINITY HQ00687 analyzer (ABBOTT, Hannover, German) (Chemiluminescent Microparticle Immunoassay, CMIA), whereas the measurements for glucose, creatinine, uric acid, total cholesterol, LDL cholesterol, triglycerides, calcium, magnesium, AST, and total proteins were performed using the CMIA technique using Abbott ALINITY AC06028 (ABBOTT, Hannover, German).

We used the annual averages of these values for each patient individually. We mention that the tacrolimus blood concentration values were routinely evaluated at six months or in the context of graft dysfunction for all patients at the Renal Transplant Institute, and the dosages were eventually modified accordingly.

We used the WHO definition of NODAT, namely, fasting glycemic values over 126 mg% in at least three different measurements throughout monitoring. Additionally, the values for HbA1c were collected to confirm the diagnosis of NODAT.

### 4.4. Tacrolimus Formulations and Dosing Protocols

This study aims to evaluate two important coordinates regarding the consequences of post-transplant TAC administration. Starting from the premise of different bioavailabilities of this drug, we performed the TAC dissolution test at different pH values and at different post-administration time intervals. Subsequently, we measured the TAC level resulting from the use of immediate-release and slow-release pharmaceutical forms. Subsequently, we sought to integrate the in vitro findings with clinical outcomes by analyzing the incidence of NODAT in the monitored patient cohort treated with tacrolimus.

#### 4.4.1. Dissolution Test

Given that the TAC level in blood is dependent on the release rate from the pharmaceutical form, we performed testing of the TAC level that is released from the pharmaceutical forms used by the analyzed renal transplant patients at different time intervals and pH values of the dissolution medium. In this regard, we analyzed TAC release from PROGRAF capsules (Astellas Ireland Co., Ltd., County Kerry, Ireland), ADVAGRAF (Astellas Ireland Co., Ltd., County Kerry, Ireland), and TACROLIMUS (Sandoz-Lek Pharmaceuticals d.d., Ljubljana, Slovenia). Regarding the pH of the solution, we chose as the dissolution medium for the studied pharmaceutical forms aqueous solutions with pH values of 1.2 (0.1 M HCl (Merk, Darmstadt, Germany)), pH 4.5 (acetate buffer) (Merk, Darmstadt, Germany), and pH 6.8 (phosphate buffer) (Merk, Darmstadt, Germany) to monitor possible differences that may appear as a result of the consumption of medications associated with these patients’ therapy and foods that modify the pH of digestive juices and influence TAC bioavailability and implicitly the risk of other conditions dependent on TAC blood level. The analyzed samples are TAC capsules (5 mg) that come from open-circuit pharmacies. In order to determine the disaggregation time, we used an Electrolab TDT-08L (USP) device consisting of 8 glass tubes with a capacity of 1000 mL each and a thermostatic bath at a temperature of 37 ± 0.5 °C. Each tube is equipped with a vertical metal rod that rotates at 30 rpm in 300 mL of liquid medium from each tube in which the analyzed samples were introduced. The dissolution test is considered appropriate when the residue in each tube does not present a palpable core, being made up of a soft mass. In order to determine the dissolution time of the analyzed samples, distilled water at different pH values, prepared according to the formula, was used as a dissolution medium. At intervals of 15 min, 30 min, 60 min, 90 min, 120 min, and 360 min, samples representing 5 mL from each tube were taken and immediately replaced with the same volume from the dissolution medium.

#### 4.4.2. Tacrolimus Dosing

A TAC stock solution, a reference substance, was prepared in acetonitrile (Merk, Darmstadt, Germany) at a concentration of 2 mg/mL (solution A). A volume of 0.5 mL from this solution was mixed with 0.5 mL of concentrated sulfuric acid (98%) (Merk, Darmstadt, Germany), a reaction that determines protonation and extension of the TAC (Sigma-Aldrich, Hamburg, Germany) conjugation system, leading to the formation of a derivative with intense absorption at 290 nm. The resulting mixture was diluted with 9 mL of distilled water, obtaining solution B, with a concentration of 100 µg/mL.

From this solution, standard solutions were prepared with concentrations of 25, 40, 50, 60, and 75 µg/mL, used for constructing the calibration curve. The absorbance of the solutions was measured at 290 nm using a UV-VIS spectrophotometer equipped with 1 cm quartz cells (UV/VIS Spectrophotometer T70 + PG Instruments Ltd, Wibtoft, UK, Soft UVWIN 5.0, MS Office 2023). As a control solution, a solution obtained by treating acetonitrile with concentrated sulfuric acid and distilled water in the same proportions, without TAC, was used.

The method showed good linearity in the 25–100 µg/mL range, with the calibration curve obtained with a correlation coefficient (R) of 0.9924. The specificity of the method was confirmed by the absence of significant interferences at 290 nm from the components of the matrix used. The validated calibration curve was applied for the quantitative determination of TAC in the analyzed samples.

#### 4.4.3. Statistical Analysis

Statistical analysis was performed using Jamovi 2.7.2. Continuous variables were presented as mean ± standard deviation (SD) and median, while categorical variables were expressed as frequencies (counts) and percentages. The independent samples Mann–Whitney U test was used for continuous variables, and the Fisher’s exact test was applied to compare categorical variables. A *p*-value < 0.05 was considered statistically significant. A multiple regression analysis was performed to assess the predictive strength of the variables potentially associated with the onset of NODAT.

## 5. Conclusions

Tacrolimus, a fundamental part of immunosuppressive therapy in renal transplant patients, presents high dose-dependent toxicity. Maintaining a minimum effective tacrolimus concentration is essential to reduce the risk of adverse effects while preserving graft function. Personalized tacrolimus therapy remains crucial for the efficacy, safety, and minimization of adverse reactions in renal transplant patients. In this study, the incidence of NODAT was high, with no significant difference between patients treated with immediate-release and extended-release tacrolimus formulations. Advanced age negatively influenced NODAT occurrence in patients treated with extended-release tacrolimus, particularly in those with impaired renal graft function and a higher prevalence of dyslipidemia and arterial hypertension.

Although differences in dissolution profiles were identified between formulations, these did not translate into significant differences in NODAT incidence, suggesting that additional determinants such as genetic polymorphisms, drug–drug interactions, and concomitant therapies may play an important role. The single-center, pilot nature of this study and the limited sample size represent inherent limitations, and further multicenter investigations using validated analytical methods are needed. The integration of pharmacogenetic and pharmacokinetic monitoring strategies may support a more individualized tacrolimus therapy and contribute to reducing the burden of NODAT in renal transplant recipients.

The complex interplay between pharmaceutical formulation characteristics, individual patient-specific factors, and clinical outcomes emphasizes the necessity for personalized approaches to immunosuppressive therapy in renal transplant recipients.

## Figures and Tables

**Table 1:** Analyzed samples.

Product Name	Advagraf (Capsules)	Tacrolimus (Capsules)	Prograf (Capsules)
Active substance	Tacrolimus monohydrate	Tacrolimus monohydrate	Tacrolimus monohydrate
Excipients	Hypromellose Ethylcellulose Lactose monohydrate Magnesium stearate	Hypromellose Croscarmellose sodium Lactose monohydrate Magnesium stearate	Hypromellose Croscarmellose sodium Lactose monohydrate Magnesium stearate
Release mode of active substance	Extended	Immediate	Immediate

**Table 2:** TAC release rate (%) from pharmaceutical forms.

Time	Prograf			Tacrolimus			Advagraf		
	pH 1.2	pH 4.5	pH 6.8	pH 1.2	pH 4.5	pH 6.8	pH 1.2	pH 4.5	pH 6.8
15 min	38%	35%	30%	36%	30%	29%	5%	4%	3%
30 min	66%	62%	60%	65%	60%	58%	8%	6%	5%
60 min	83%	81%	80%	80%	78%	78%	20%	18%	18%
90 min	85%	83%	80%	82%	80%	80%	35%	33%	33%
120 min	95%	93%	92%	91%	90%	90%	40%	40%	40%
360 min	100%	100%	100%	100%	100%	100%	45%	46%	48%

**Table 3:** Comparative findings in the patients treated with IR-TAC and ER-TAC.

Patients Characteristics M ± SD/Median	Entire Cohort n = 66	IR-TAC n = 33	ER-TAC n = 33	*p*
Age (years)	50.273 ± 10.14/50	50.121 ± 11.683/49	50.424 ± 8.511/50	0.905
Gender (female)	31 (47%)	17 (25.8%)	14 (21.2%)	0.62
Tx vintage	14 ± 6.919/12.5	14.365 ± 5.079/14	13.636 ± 8.437/10	0.673
BMI	26.285 ± 3.503/26.15	26.709 ± 3.013/26.2	25.861 ± 3.934/25.3	0.329
Cadaveric donor	52 (78.8%)	28 (84.84%)	24 (72.72%)	0.366
Documented acute rejection	16 (24.2%)	8 (12.1%)	8 (12.1%)	0.773
Corticotherapy	60 (90.9%)	33 (100%)	27 (81.81%)	0.024
TAC mean dose	3.97 ± 1.637/4	4.227 ± 1.663/4	3.727 ± 1.596/4	0.217
Creatinine	1.468 ± 0.686/1.25	1.515 ± 0.807/1.3	1.421 ± 0.549/1.2	0.579
eGFR (mL/min)	61.98 ± 28.045/59	61.697 ± 30.424/61	62.273 ± 25.93/57	0.934
Glucose	96.318 ± 20.795/90	95.303 ± 18.437/90	97.333 ± 23.159/87	0.695
Hb A1c	5.668 ± 0.720/5.45	5.7 ± 0.663/5.9	5.573 ± 0.744/5.2	0.723
NODAT	27 (40.9%)	13 (39.39%)	14 (42.42%)	1
hs-CRP	4.814 ± 4.192/3.5	4.773 ± 4.137/3.5	4.855 ± 4.31/3.5	0.938
AST	29.698 ± 39.53/23	24.406 ± 8.257/23	35.161 ± 55.67/23	0.620
T cholesterol	188.11 ± 46.998/191.5	179.077 ± 38.428/180	196.5 ± 53.075/209	0.176
Triglycerides	156.354 ± 50.059/160	150.606 ± 54.006/143	162.281 ± 45.73/170	0.351
Calcium	8.808 ± 1.176/8.9	8.887 ± 0.802/8.9	8.718 ± 1.506/8.95	0.582
Magnesium	1.697 ± 0.158/1.7	1.686 ±0.162/1.7	1.708 ± 0.156/1.7	0.569
Uric acid	6.395 ± 1.277/6.7	6.512 ± 1.002/7	6.267 ± 1.532/6.65	0.451
Proteinuria g/24 h	0.656 ± 0.838/0.425	0.536 ± 0.765/0.32	0.775 ± 0.902/0.5	0.251

*t* test continuous variables, x2/Fisher test categorical variables.

**Table 4:** Comparative findings in the patients treated with and without NODAT.

Patients Characteristics M ± SD/Me	NODAT n = 27	Non-NODAT n = 39	OR (Univariable)	*p*
Age (years)	49.111 ± 8.541/50	51.077 ± 11.155/51	1.00 (0.94–1.06, *p* = 0.881)	0.583
Gender (female)	13 (48%)	18 (46.15%)	1.59 (0.51–5.01, *p* = 0.423)	0.873
BMI	27.619 ± 3.459/27.3	25.362 ± 3.266/24.8	1.20 (1.01–1.45, *p* = 0.047)	0.008
Tx vintage	10.519 ± 3.469/10	16.410 ± 7.687/16	0.97 (0.89–1.04, *p* = 0.414)	0.070
Cadaveric donor	21 (77.77%)	31 (79.48%)		0.867
Documented acute rejection	6 (22.22%)	10 (25.64%)	0.99 (0.26–3.48, *p* = 0.990)	0.750
Corticotherapy	25 (92.59%)	35 (89.74%)	1.74 (0.21–36.67, *p* = 0.642)	0.692
TAC mean dose	3.593 ± 1.494/3.5	4.244 ± 1.697/4	0.70 (0.46–1.01, *p* = 0.072)	0.147
ADV	14 (51.85%)	19 (48.71%)		0.802
TAC	13 (48.14%)	20 (51.28%)		0.802
Creatinine	1.481 ± 0.636/1.3	1.459 ± 0.627/1.2	0.73 (0.27-.73, *p* = 0.498)	0.615
eGFR (mL/min)	60.222 ± 26.56/57	63.205 ± 29.308/62	1.00 (0.98–1.02, *p* = 0.943)	0.730
Glucose	113.296 ± 22.35/115	84 ± 7.155/84	1.12 (1.06–1.1, *p* < 0.001	<0.001
HbA1c	6.326 ± 0.43/6.3	5.159 ± 0.381/5.1		<0.001
hs-CRP	5.867 ± 5.197/4	4.085 ± 3.20/3	1.13 (0.98–1.34, *p* = 0.111)	0.112
AST	26.480 ± 9.55/24	24 ± 8.829/23	1.05 (0.98–1.14, *p* = 0.199	0.569
T cholesterol	201.631 ± 50.81/219	180.771 ± 43.804/190	1.01 (1.00–1.03, *p* = 0.103)	0.118
LDL cholesterol	126.37 ± 49.65/115	114.49 ± 34.104/116	1.01 (1–1.03, *p* = 0.074)	0.447
Triglycerides	184.111 ± 42.95/189	136.632 ± 45.563/130	1.03 (1.01–1.05, *p* = 0.001)	<0.001
T proteins	6.938 ± 0.404/6.9	6.663 ± 1.184/6.9	1.44 (0.73–4.84, *p* = 0.456)	0.624
Calcium	8.7 ± 1.52/8.9	8.886 ± 0.868/8.9	0.87 (0.51–1.39, *p* = 0.539)	0.976
Magnesium	1.618 ± 0.148/1.6	1.752 ± 0.143/1.74	0.01 (0.00–0.37, *p* = 0.030)	<0.001
Uric acid	6.319 ± 1.705/7	6.449 ± 0.886/6.7	0.82 (0.52–1.27, *p* = 0.380)	0.594
Proteinuria g/24 h	0.883 ± 0.73/0.6	0.498 ± 0.876	1.66 (0.89–3.29, *p* = 0.115)	<0.001

Mann–Whitney test, Fisher test.

**Table 5:** Comparative findings in the patients treated with long release vs. immediate release.

NODAT patients Characteristics M ± SD/Median	ER-TAC n = 14	IR-TAC n = 13	*p*
Age (years)	51.786 ± 5.563/50	46.231 ± 10.35/47	0.103
Gender (female)	7 (50%)	6 (46.15%)	1
BMI	28.35 ± 3.6/28.45	26.831 ± 3.255/26.2	0.174
Tx vintage	8.429 ± 2.344/8	13.077 ± 3.095/13	<0.001
Cadaveric donor	7 (50%)	3 (23.07%)	0.236
Documented acute rejection	3 (21.42%)	3 (23.07%)	1
Corticotherapy	12 (85.71%)	13 (100%)	0.481
TAC mean dose	3.143 ± 1.365/3	4.132 ± 1.770	0.213
Creatinine	1.512 ± 0.574/1.3	1.448 ± 0.718/1.3	0.697
eGFR (mL/min)	56 ± 24.019/55.5	64.769 ± 29.329/61	0.512
Glucose	116 ± 24.58/120.5	110.385 ± 20.259/110	0.436
HbA1c	6.457 ± 0.386/6.4	6.346 ± 0.35/6.3	0.338
hs-CRP	6,621 ± 5.535/4.8	5.054 ± 4.893/4	0.243
AST	29.308 ± 11.056/28	23.417 ± 7.948/20.5	0.157
T cholesterol	207.636 ± 61.74/210	193.375 ± 32.53/200	0.431
Triglycerides	196.429 ± 26.795/191	170.846 ± 53.412/167	0.174
Calcium	8.467 ± 2.016/8.95	8.915 ± 0.891/8.7	0.604
Magnesium	1.614 ± 0.143/1.6	1,622 ± 0.159/1.64	0.921
Uric acid	6.2 ± 2.146/7	6.438 ± 1.192/7	0.857
Proteinuria g/24 h	1.105 ± 0.695/0.85	0.643 ± 0.736/0.45	0.003
Insulin	5 (35.71%)	1	0.164
Oral antidiabetics	4	5	0.694
Diet	4	7	0.251

Mann–Whitney test, continuous variables; Fisher test, categorical variables.

**Table 6:** Model (diabetes mellitus) coefficients.

						95% Confidence Interval	
Predictor	Estimate	SE	Z	*p*	Odds Ratio	Lower	Upper
Age	0.068	0.073	0.941	0.347	1.071	0.929	1.234
Gender (female)	−0.407	1.336	−0.305	0.760	0.665	0.048	9.132
Deceased donor	−0.086	1.440	−0.060	0.952	0.918	0.055	15.428
Tx vintage	−0.291	0.142	−2.048	0.041	0.748	0.566	0.988
Acute rejection:	2.566	1.850	1.387	0.165	13.009	0.347	488.337
ER tacrolimus	−0.027	1.265	−0.021	0.983	0.974	0.082	11.612
Corticotherapy	1.435	6.244	0.230	0.818	4.199	0.000	866,526.459
eGFR	0.014	0.027	0.524	0.601	1.014	0.963	1.068
BMI	0.205	0.232	0.884	0.377	1.228	0.779	1.935
hs CRP	−0.145	0.178	−0.815	0.415	0.865	0.611	1.225
T cholesterol	−0.021	0.018	−1.153	0.249	0.979	0.945	1.015
Triglycerides	0.052	0.021	2.449	0.014	1.053	1.010	1.098
Magnesium	−7.275	6.301	−1.155	0.248	0.001	0.000	159.894
Uric acid	−0.934	0.600	−1.558	0.119	0.393	0.121	1.273
Proteinuria	0.532	0.765	0.696	0.487	1.703	0.380	7.625

Legend: Tx—transplant, ER—extended release, BMI—body mass index, hs-CRP—highly sensitive C-reactive protein.

**Table 7:** Odds Ratio for the main potential NODAT predictors.

Dependent: DM Mean (SD)	Non-NODAT	NODAT	OR (Univariable)	OR (Multivariable)
Age	50.0 (11.2)	49.6 (7.3)	1.00 (0.94–1.06, *p* = 0.881)	1.07 (0.94–1.26, *p* = 0.347)
Gender (female)	14 (58.3)	10 (41.7)	1.59 (0.51–5.01, *p* = 0.423)	0.67 (0.04–9.69, *p* = 0.760)
DD	27 (64.3)	15 (35.7)	0.97 (0.25–4.21, *p* = 0.968)	0.92 (0.05–21.96, *p* = 0.952)
Tx VINTAGE	16.4 (8.1)	10.6 (3.5)	0.83 (0.71–0.94, *p* = 0.009)	0.75 (0.54–0.95, *p* = 0.041)
Acute rejection	9 (64.3)	5 (35.7)	0.99 (0.26–3.48, *p* = 0.990)	13.01 (0.46–1297.03, *p* = 0.165)
ER tacrolimus	16 (59.3)	11 (40.7)	1.55 (0.50–4.93, *p* = 0.450)	0.97 (0.07–13.83, *p* = 0.983)
Corticotherapy	31 (63.3)	18 (36.7)	1.74 (0.21–36.67, *p* = 0.642)	4.20 (0.00–102,701.30, *p* = 0.818)
eGFR	61.1 (29.5)	61.6 (26.5)	1.00 (0.98–1.02, *p* = 0.943)	1.01 (0.96–1.08, *p* = 0.601)
BMI	25.3 (3.4)	27.4 (3.4)	1.20 (1.01–1.45, *p* = 0.047)	1.23 (0.75–1.96, *p* = 0.377)
CRP	3.9 (3.2)	5.9 (5.1)	1.13 (0.98–1.34, *p* = 0.111)	0.87 (0.55–1.22, *p* = 0.415)
T-chol	179.3 (43.5)	201.6 (50.8)	1.01 (1.00–1.03, *p* = 0.103)	0.98 (0.94–1.01, *p* = 0.249)
Triglyceride	140.3 (43.9)	191.5 (38.3)	1.03 (1.01–1.05, *p* = 0.001)	1.05 (1.02–1.12, *p* = 0.014)
Magnesium	1.7 (0.1)	1.6 (0.2)	0.01 (0.00–0.37, *p* = 0.030)	0.00 (0.00–14.01, *p* = 0.248)
Uric acid	6.5 (0.9)	6.1 (1.9)	0.82 (0.52–1.27, *p* = 0.380)	0.39 (0.08–1.05, *p* = 0.119)
Proteinuria	0.6 (0.9)	1.0 (0.8)	1.66 (0.89–3.29, *p* = 0.115)	1.70 (0.39–9.50, *p* = 0.487)

## Data Availability

The original contributions presented in the study are included in the article, further inquiries can be directed to the corresponding author.
